# REHABS: An Innovative and User-Friendly Device for Rehabilitation

**DOI:** 10.3390/bioengineering11010005

**Published:** 2023-12-21

**Authors:** Patrizia Vizza, Nicola Marotta, Antonio Ammendolia, Pietro Hiram Guzzi, Pierangelo Veltri, Giuseppe Tradigo

**Affiliations:** 1 Department of Medical and Surgical Sciences, University of Catanzaro Magna Graecia, 88100 Catanzaro, Italy; ammendolia@unicz.it (A.A.); hguzzi@unicz.it (P.H.G.); 2 Department of Clinical and Experimental Medicine, University of Catanzaro Magna Graecia, 88100 Catanzaro, Italy; nicola.marotta@unicz.it; 3 DIMES Department, University of Calabria, 87036 Rende, Italy; pierangelo.veltri@dimes.unical.it; 4 Department of Theoretical and Applied Sciences, University e-Campus, 22060 Novedrate, Italy; giuseppe.tradigo@uniecampus.it

**Keywords:** rehabilitation, medical device, follow-up, monitoring

## Abstract

Rehabilitation is a complex set of interventions involving the assessment, management, and treatment of injuries. It aims to support and facilitate an individual’s recovery process by restoring a physiological function, e.g., limb movement, compromised by physical impairments, injuries or diseases to a condition as close to normal as possible. Innovative devices and solutions make the rehabilitation process of patients easier during their daily activities. Devices support physicians and physiotherapists in monitoring and measuring patients’ physical improvements during rehabilitation. In this context, we report the design and implementation of a low-cost rehabilitation system, which is a programmable device designed to support tele-rehabilitation of the upper limbs. The proposed system includes a mechanism to acquire and analyze data and signals related to rehabilitation processes.

## 1. Introduction

Physiotherapy aims to help patients improve their movements and function, and reduce pain through exercise, manual or electro-therapy and also education (i.e., physiotherapists inform patients about their condition and how to manage it). Physiotherapy focuses on rehabilitation and prevention by helping to restore motor functionalities and preventing future complications [1,2,3,4].

We focus on the problem of efficiently supporting both physicians and patients in designing and performing population-scale rehabilitation exercises and acquiring relevant data [5,6].

Physiotherapy treats acute or chronic pain and physical impairment caused by injury, trauma, disease, or disability. Integrating physical activities promotes significant changes in improving life quality of patients with chronic and progressive disabilities [7,8,9]. Functional neurological disorders (FND) are among the most common causes of neurological disability and physical therapies have been recognized to play an important role in the treatment of these disorders [10,11,12,13,14]. Motor rehabilitation strategies can be associated with innovative technologies to help the patient regain normal control of movement. In [15] a longitudinal case study, a design has been proposed to track recovery of motor function after severe traumatic brain injuries, through a multimodal neuroimaging approach. The authors in [16,17] provide an overview of the positive effects of physical activity on Parkinson’s disease. Neurological and musculoskeletal diseases such as arthritis, cerebral palsy, Parkinson’s disease, and stroke affect hand functions. In this context, manual dexterity is the capability to manage the movements of the upper limb, hand and fingers, developed through learning, training and experience, and assessed via speed and accuracy evaluation of movement [18]. Hand impairment could be recovered by performing well-established physical therapy. Finally, kinesio-therapic intervention can be used to recover hand functionality, lost or impaired by chronic diseases [19].

Physiotherapy represents an important step in patient management, in combination with examination, diagnosis, physical intervention, and patient education.

Rehabilitation aims to help the patient recover and maintain normal movement by following standard well-defined protocols, often using medical devices [20,21,22]. These devices allow clinicians to collect useful data for the monitoring, control, and management of the recovery process. For example, Ref. [23] reports the same general-purpose indication of the rehabilitative effects of soft robotics in hand dysfunction. Chu et al. in [23] reported that recently has seen a rapid growth in soft robotic device development for hand-fingers rehabilitative approach; in fact, proof-of-concept prototypes show a wide series of technical solutions, nevertheless further implementations are necessary to be done in actuator design, safety, and clinical applications to advance to clinical scenario. Finally, robotic exoskeletal platforms support the recovery of upper and lower limbs functions in patients with motor impairment, especially in neuro-rehabilitation [24,25].

Internet of Things (IoT) allows for easier and more efficient patient telemonitoring, by (i) allowing for remote monitoring of specific clinical parameters, (ii) helping to identify abnormally acquired values, or (iii) detecting wrongly performed procedures during the rehabilitation phase [26]. Telemonitoring is particularly useful when patients live far from the clinical structure or in the case of chronic patients who cannot move from their homes. It can also be used as a “continuity of care” service following discharge from hospital. Such a situation arose recently, during the COVID-19 pandemic, when access to clinical structures was severely limited [27]. Telemonitoring is also useful in clinical pathways (CPWs), where collecting large amounts of data allows for faster and more accurate diagnosis of disease as well as in the screening phases, where analyses are performed to optimize costs of patient hospitalization [28,29]. However, portable medical devices for telemonitoring can cause inconvenience for the patient, for example, when they get in the way of daily activities or malfunctions occur that cannot be solved easily [30]. Multiple home rehabilitation devices are available and implemented; however, some technologies face limitations in the application, availability, as well as cost and preparation of appropriate rehabilitation therapist to support the rehabilitation process [31]. Arntz et al. in [32] recently demonstrated that feasible and effective approaches are needed to implement functional home-based recovery, which meet patients’ demands and ensure adequate levels of treatment.

Many devices have been used for applied research in rehabilitation procedures [33,34]. In [35], the authors present a miniaturized version of a hospital device for hand rehabilitation in post-stroke hemiplegic patients. In [36,37] portable and wearable hand exoskeleton systems for exercising in movement rehabilitation have been developed.

In this paper we present the design and implementation of a rehabilitation system for the recovery of hand impairment caused by neurological or musculo-skeletal disorders. We designed and implemented a hardware and software tool for acquiring biometric signals. This system can help subjects affected by hand dysfunction in their rehabilitation in a simple and non invasive way. Compared to other available devices, it does not include wearable parts, i.e., parts that need replacing. Moreover, it does not require an expensive setup and can be easily used for remote rehabilitation. Exercises can be designed for different diseases related to hand disabilities (e.g., patients affected by neurodegenerative disorders or strokes).

## 2. Materials and Methods

The proposed contribution results from a synergistic activity carried out by the Physical and Rehabilitation Medicine Unit, the Bioengineering unit of the Magna Graecia University of Catanzaro and the SMARTEST Laboratory at eCampus University. We present a system to support physicians and patients in designing, executing, and monitoring rehabilitation processes. Patients at home can use it, and the data is collected into a cloud-based platform to be integrated into an electronic patient record database. The system comprises an Arduino-based device and a health informatics module that can guide personalised rehabilitation exercises. The system includes a pressure sensor to acquire and measure the force applied by the finger on rehabilitation plates and two ultrasonic sensors capable of guiding the position of the finger during exercises. The device has been designed to improve the fine mobility of the upper limb through targeted exercises. The data collected by the device during the exercises provide useful information for physicians in monitoring treatments, which specialists can then use to model the patient’s health status. A preliminary version of the architecture is reported in [22]. Experiments and tests have been performed at the University Magna Graecia Clinical Hospital. We here report the architecture, configuration, setting, implementing exercises, and platform.

### 2.1. System Architecture

**Figure 1:**
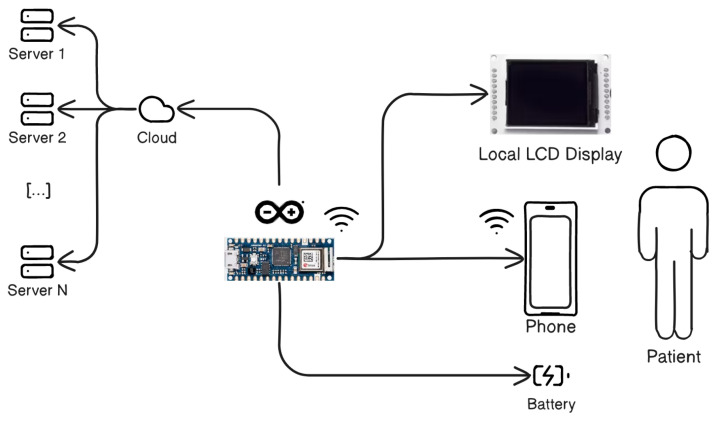
System architecture. An Arduino-compatible board based on ESP32 is used with built-in wifi for cloud connectivity with the server, for LCD display and phone contact with the patient.

The architecture of the designed system is shown in Figure 1.

It comprises a board with a new Arduino-compatible board based on ESP32 with built-in wifi connectivity, enclosed in a custom-designed and 3D-printed external case. The system acquires patient input through two capacitive sensors, the use of which can be decided during the exercise definition phase, an LCD display for status, an SD Card drive for data logging, status LEDs and buttons for basic interaction. the system includes remote data memorization for telemonitoring and touch sensors to acquire pressure and detect finger position, allowing physicians to analyze and monitor the progress of the hand rehabilitation. Moreover, the Arduino-based device includes an external memory (an SD Card) allowing data storage. LED signals are used to monitor the functionality of the correct activity.

The LCD display interfaces with the Arduino microcontroller via I2C serial communication protocol. It is a backlit display which shows different messages (e.g., *initialization* when the system is connected to the computer via USB, *exercises* when the device starts up correctly and the list of the proposed exercises is proposed, and *calibration* when the system is in the calibration phase). Phase must be performed when external conditions could modify the measurement. To perform the calibration, the pad must be touched for few seconds following the instructions shown on the LCD display. This procedure increases the accuracy and the precision of the device and the calibration values are saved in the EEPROM memory. A set of pre-defined exercises is stored and can be selected using a simple user interface.

The capacitive sensors detect the presence of any conductive material on the plates. A voltage is applied to the four corners of the platform, which is propagated uniformly over the entire surface due to the oxide metal. When the hand (or in general any conductive material) comes into contact with the sensor surface, the surface capacitance varies and the sensor oscillates. The calibration phase implies that the capacity increasing is associated to a “touch” event; when the hand moves away, capacity decreases and it is associated with an “untouch” event. Whenever a touch or release event is detected, the external LED lights up.

**Figure 2:**
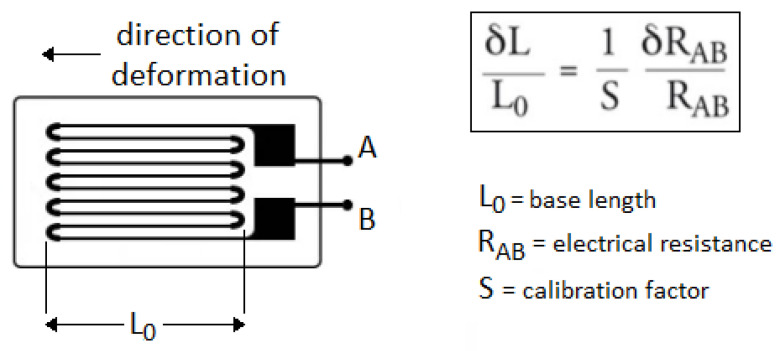
Strain gauge sensor. The schema of the sensor with the serpentine pattern and the equation representing the relation between deformation and resistance values are shown on the left and the right of the figure, respectively.

FSR402 (Force Sensing Resistor) thin-film pressure sensor has been chosen to enable the detection of physical pressure, compression and weight, with high sensitivity and accuracy to minimize acquisition error, in a simple and low cost way. It is a strain gauge sensor type consisting of a very thin conductor (e.g., nickel alloy film) arranged in a serpentine pattern on a very thin insulating substrate, as shown in Figure 2 [38]. The latter transmits to the film a deformation causing a change in length and resistance. Moreover, the strain is interpreted to determine the weight of an object placed on the sensor.

The system uses a FSR402 sensor consisting of a wire resistance strain gauge, which when subjected to stress varies its electrical resistance as R=P·L/S , where (i) *P* is the resistivity of the material ( Ω· m), (ii) *L* is the length of the resistor (m) and (iii) *S* is the section (m^2^) measures as $S=\pi \cdot D^{2}/4$ with *D* the wire diameter.

**Figure 3:**
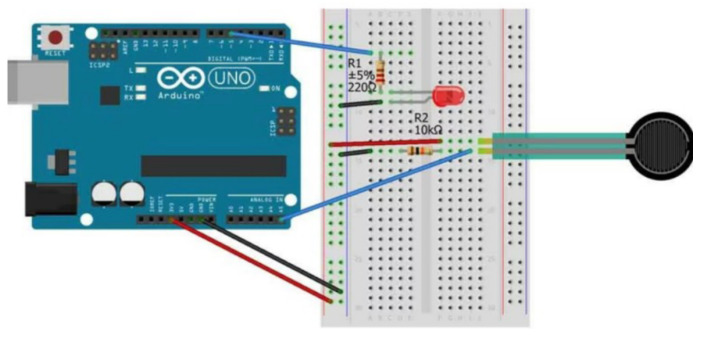
Connection of the FSR402 sensor with related electrical components to the Arduino board.

This sensor was integrated into the system and calibrated. To enable this sensor to support data collection during the execution of rehabilitation exercises, the following steps were considered: (i) detection of sensor data; (ii) calibration of the sensor with respect to a statistical measurement standard and checking its linearity; (iii) conversion of the values acquired by the sensor into kilograms; (iv) evaluation of static features (e.g., sensitivity, resolution, repeatability, stability, linearity), (v) calculation of the bias, standard deviation and variance of the measurement. To detect the sensor data, FSR402 was assembled with the Arduino board according to the scheme reported in Figure 3.

Detected FSR402 values are converted from Volt (V) to kilogram (kg) by means of a calibration procedure. Three different samples of known weight of 500 (*S*1), 1000 (*S*2) e 2000 (*S*3) grams, respectively, have been used during the procedure. The values detected by the sensor w.r.t. the three input samples and the related mean values are reported in Table 1.

**Figure 4:**
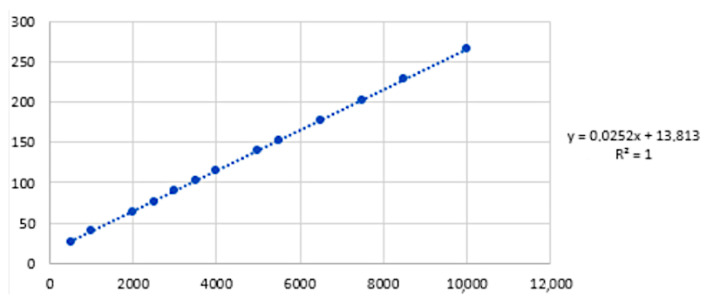
Linear calibration curve expressed in grams based on the interpolating curve that best approximates to a distribution of (*Sample, Detected value*) pairs. On the right of the figure, the equation of the conversion from Volts to grams is shown.

The linear regression gave the calibration curve expressed in grams, shown in Figure 4.

**Figure 5:**
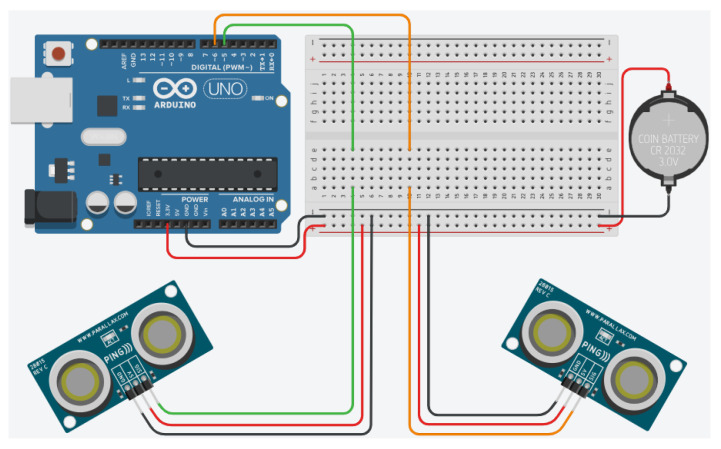
Setup of the two ultrasonic sensors used to triangulate the position. Wires indicated in red are connected to Vcc (+5V) and black are connected to ground, while other colors are signals (0–5 V).

The system uses an ultrasonic sensor (HC-SR04, see Figure 5) to detect the location of an object by measuring the distance between each sensor and the object (e.g., patient’s finger). Ultrasonic sensors are based on the time-of-flight measurement of an ultrasound wave travelling between the sensor and the object to be detected. Since the sound waves propagate at the speed of 343.8 m/s (at 20 °C), we calculate the distance between the sensor and the object with: d=(w·T)/2[m] (note that distance *d* is travelled twice by the wave).

**Figure 6:**
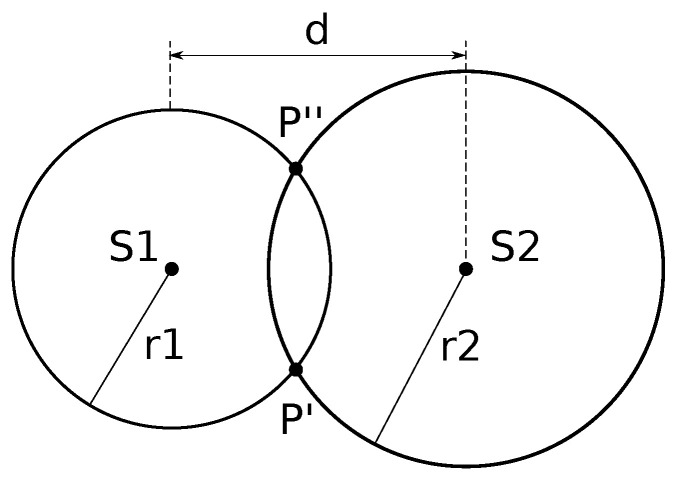
Schema of the two ultrasonic sensors (S1 and S2), mounted at distance d, to detect the position of an object. Sensor S1 will detect a distance of r1, while S2 a distance of r2. By combining the two pieces of information we can calculate the solution.

In order to detect the position of the patient’s finger which interacts with the capacitive sensor, we use two ultrasonic sensors (see Figure 5). Each ultrasonic sensor will detect the distance from the object and this information can be combined to calculate the position of the object with respect to the device. The two ultrasonic sensors, indicated with S1 and S2 in Figure 6, placed at distance d, measure the distances r1 and r2 at which the object is detected respectively.

Algorithm 1 reports the pseudo-code used by the device to combine data from the ultrasonic sensors.
**Algorithm 1** Object detection algorithm**Require:** *d* distance between the two sensors, 
$t_{1}$
 time of flight from sensor S1, 
$t_{2}$
 time of flight from sensor S2 1: /* Convert time of flight from sensors to distances */ 2: 
$r_{1}\leftarrow 0.03438*t_{1}/2$
3: 
$r_{2}\leftarrow 0.03438*t_{2}/2$
4: /* Combine to calculate object coordinates */ 5: 
$x\leftarrow \left(r_{2}^{2}-d^{2}-r_{1}^{2}\right)/\left(2d\right)$
6: 
$y\leftarrow \sqrt{r_{1}^{2}-x^{2}}$
7: **return**

x,y


### 2.2. Device Use Configuration

This is a proof-of-concept design and implementation study. Given the non-pharmacol-ogical, non-invasive and non-experimental nature of the study, the Institutional Review Board did not deem it necessary to register and issue a code. However, the study was conducted in accordance with the ethical principles for medical research involving human subjects outlined in the Declaration of Helsinki. All participants were fully informed about all procedures and eventually voluntarily agreed to participate in the study. Participants were recruited from the Physical and Rehabilitative Unit of the University Hospital “Renato Dulbecco” of Catanzaro, Italy. Inclusion criteria were: (1) 18–70 years of age; (2) no cognitive impairment (Mini-Mental Status Examination ≥24 . We excluded patients with the following characteristics: (1) acute upper limb impairment (trauma, infections or acute orthopedic or neurological disorders); (2) drugs that may affect manual dexterity administered in the last 4 weeks; (3) documented history of seizures and brain aneurysms. After enrolment, all study participants were screened using the most common and accessible manual dexterity assessment tool available: the Nine-Hole Peg Test (NHPT), a score capable of being administered by asking the participant to take the pegs from a container, one by one, and place them into holes on the board as quickly as possible. Therefore, the subjects were assigned to a control group with an NHPT ≤30 s or to an experimental group with an NHPT score >30 s, in order to test the evaluation performance of the device for different degrees of fine motility [39].

Before use, the device has to be set up and calibrated w.r.t. the patient. Communication parameters are set before exercises can be both run for the individual patient. Then for each patient, exercises can be run and stored locally and in the cloud-based server.

The user chooses the exercise to be performed and the device records the touches storing data such as (i) exercises and duration; (ii) starting and ending time interval; (iii) events (e.g., touch or release).

### 2.3. Rehabilitation Exercises

The proposed rehabilitation exercises aim to: (i) measure the sensorimotor ability of the upper or lower arm and (ii) improve its fine mobility. The first exercise type (E1) is used to evaluate hand coordination. The second (E2) is used to evaluate the ability to perform precision movements in a short time. The third (E3) measures the ability to rotate the wrist.

E1 consists in performing repeated interactions on the two capacitive sensors with a given frequency (e.g., 60 bpm, 120 bpm), by alternating taps on the two sensors. The subject must perform one *touch* gesture (T) per second and continue the exercise for a given time interval (e.g., E1 for 30 s). The time for each touch event is calculated by subtracting two consecutive *touch* and *un-touch* timestamps.

**Figure 7:**
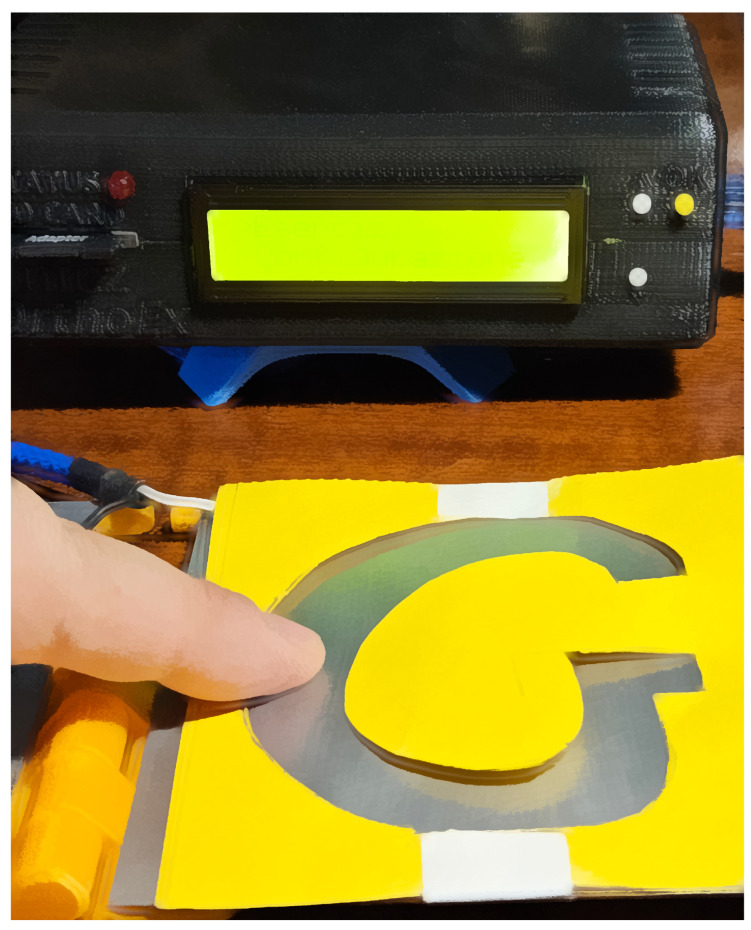
E2 exercise type in which the subject follows the shape of a letter (in the shown example the letter G) without loosing contact with the capacitive sensor (metal part).

E2 consists in following letter shapes with the finger. The subject must trace the letters in the shortest time possible. The letters used for rehabilitation excercises are: *I*, *V*, *M* and *G*. An example of the letter *G* drawing is reported in Figure 7.

**Figure 8:**
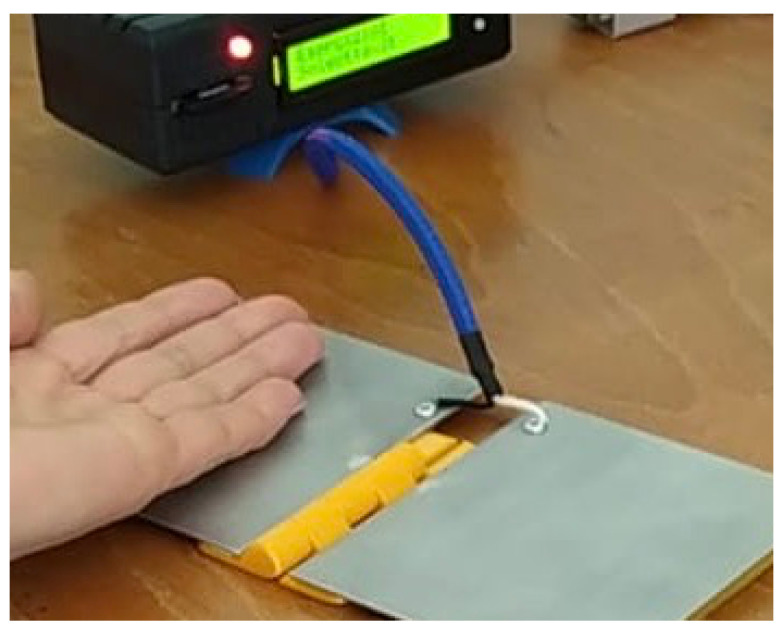
E3 exercise type in which the subject performs a prono-supination routine, alternating the touch of the capacitive sensor with the palm and the dorsal side of the hand. The subject is instructed in adopting the fastest pace within a maximum 30 s. The device will memorize the touch and untouch events and the count of the touch events in the data preprocessing phase.

E3 is also called prono-supination (P-S) exercise. It consists in touching one of the two surfaces and immediately after touching the back on the other. This movement consists of a rotation of the wrist. The exercise is performed for 30 s. An example of this exercise is shown in Figure 8.

The sensor relieves the touch on the capacity plate and the left sensor is used for the back and the right one for the palm. In these experiments, experimental tests have been guided by clinicians who supported patients in performing test to validate the correctness of single exercise.

## 3. Results

The implemented system has been tested on a dataset of 80 subjects (40 study subjects affected by hand dysfunction and 40 control subjects) enrolled at the Physical and Rehabilitation Medicine Unit of Mater Domini Catanzaro Hospital. The study subjects were affected by the diseases shown in Table 2. Control subjects present no upper limb pathologies.

The experiment involved the set of rehabilitation exercises performed by all the enrolled subjects. The results of exercises performed by control and study subjects were analyzed in order to assess the difference (deviation) between the two groups in terms of upper art sensorimotor ability. Finally, the results of two groups were statistically compared by using a Student t-test with significance level α=0.01 .

**Figure 9:**
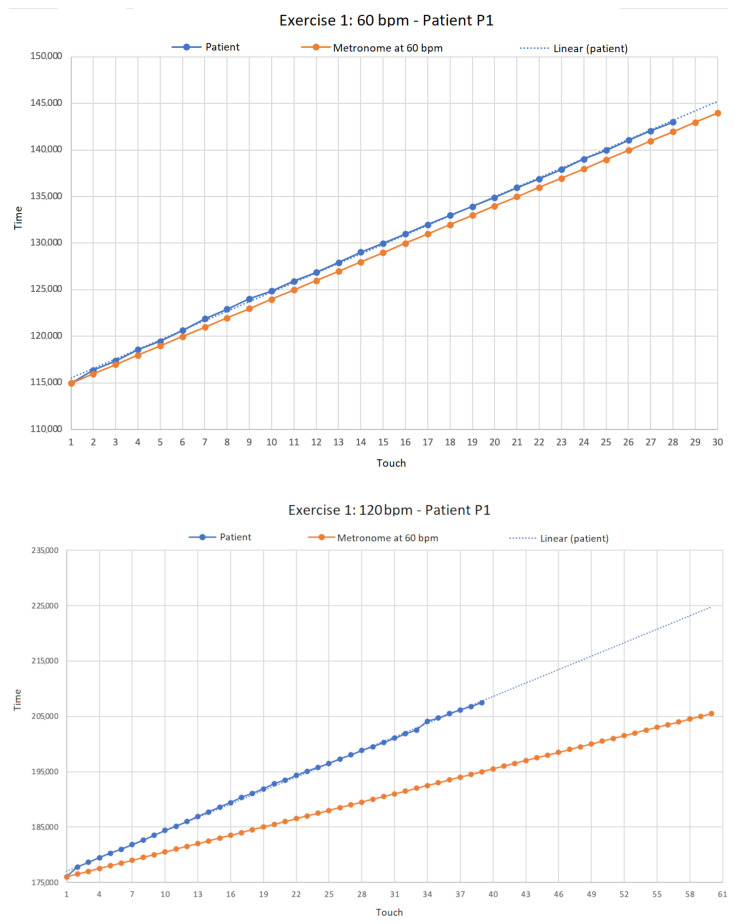
Absolute acquisition times (in milliseconds) of touch events acquired during type 1 exercise (i.e., touches on the sensor following the metronome) by patient (P1). In the upper part of the Figure we show results for metronome at 60 bpm (beats-per-minute), while in the lower part for metronome at 120 bpm.

Results for exercises, performed at 60 bpm and at 120 bpm for patient (P1) affected by Loeys-Dietz Syndrome, are reported in Figure 9.

For exercise E1 with metronome at 60 bpm, patient P1 performed 14 touches on the left capacitive sensor and 14 touches on the right one, for a total of 28 touches with an average of 591.571 ms per touch and a standard deviation of 119.858 ms. Note that the expected ideal result of the exercise should have been 30 touch events, hence, for reasons to be clinically evaluated, P1 patient completed the exercise with two touches less. Figure 9 at the top shows that the patient was unable to start in time since he misses the first touch (compare the blue bullets of the patient w.r.t. the orange bullets of the metronome). Furthermore there is an evident desynchronization with the metronome (see for instance from the seventh touch onward). In the lower graph (bottom part of Figure 9), the metronome was set to 120 bpm and patient P1 performed 19 touches on the right sensor and 20 on the left, for a total of 39 touches (compared to the expected 60), with an average of 412.692 ms for each touch and a standard deviation of 168.459 ms. With this timing of the metronome (i.e., 120 bpm), we observe that the patient experiences more difficulties in following the metronome, thereby missing more touches w.r.t. the previous case.

**Figure 10:**
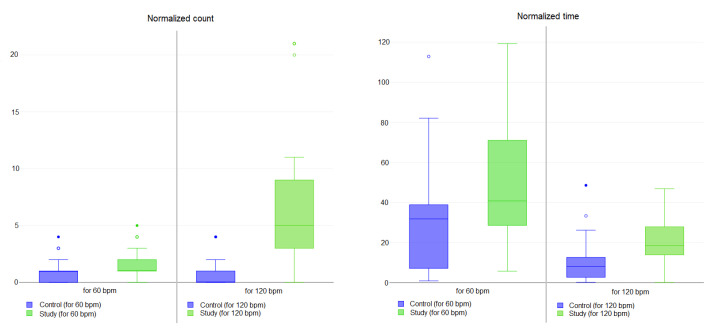
Box plot related to results for Exercise E1 for both 60 bpm and 120 bpm tasks for study (in green) and control subjects (in blue). Small circles indicate outlier data instances.

The complete results for all of the subjects analyzed (both study and control) for exercise E1 are synthesized in Table 3 and Figure 10. To normalize the data, the table reports the results expressed as the absolute value of the difference between the mean counts of T (*touch* gesture) events than the expected counts (30 T events for the metronome at 60 bpm and 60 T events for metronome at 120 bpm, respectively). This value is reported in the table as *Normalized count*. Moreover, the table also reports the absolute value of the difference between the mean time for a single T event and the expected time (1000 ms for each T event for the metronome at 60 bpm and 500 ms for each T event for metronome at 120 bpm, respectively). This value is reported in the table as *Normalized time*.

**Figure 11:**
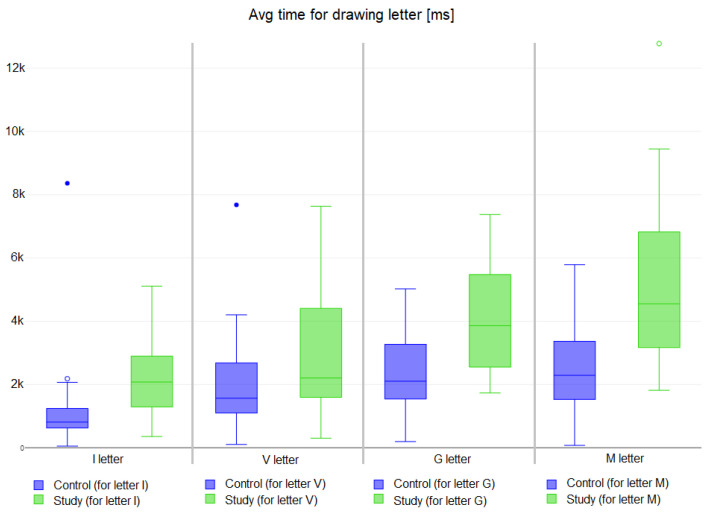
Results of Exercise E2 for drawing letters I, V, G and M for study (in green) and control subjects (in blue). Small circles indicate outlier data instances.

The results of exercise E2 are reported in Table 4 and Figure 11. Each column represents the time in milliseconds (ms) used by each subject to draw the corresponding letter (e.g., I, V, G and M) on the sensor surface. Student t-test has been applied to verify the statistical significance of the two groups, with an α value of 0.05.

**Figure 12:**
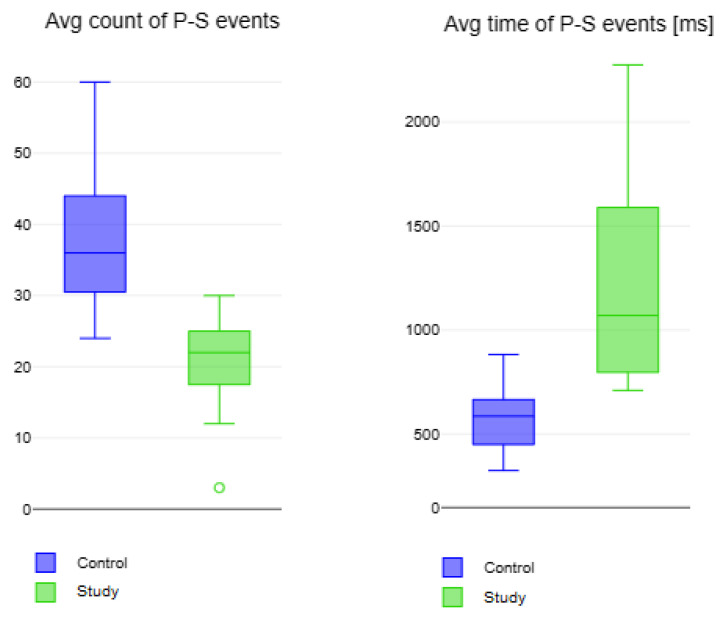
Box plot of results for Exercise E3 for the prono-supination P-S events for study (in green) and control subjects (in blue). Small circles indicate outlier data instances.

The Table 4 shows the time interval required to complete exercises. As expected, patients with hand impairment found it more difficult to draw the letters. Moreover, in some cases, the letter was drawn discontinuously (e.g., the patients were unable to maintain contact with the pad for sufficient time) or it was not drawn at all. The letter that required the most time was *M* while letter *I* was the easiest to draw. *p*-value results show a significant difference between study and control groups in drawing both *I* and *G*. For exercise E3 , the results are reported in Table 5 and Figure 12.

Patients affected by hand disability perform fewer complete rotations performed by control subjects. Also the time spent to complete the entire rotation is slower than the time of the control. *p*-values report a significant statistical difference between both parameters in the two groups.

## 4. Discussion

This proof-of-concept study aimed to evaluate the design and implementation of a low-cost rehabilitation system, i.e., a programmable device designed to support upper extremity telerehabilitation. In light of the results obtained, the device was found to be safe, reliable and supplied at a low cost. No concerns were demonstrated in the approach or in participating in the proposed exercises. Nevertheless, the reported results show that patients affected by hand disability have considerable difficulty performing the exercises and need more time to complete them. These exercises and the entire rehabilitation system could support patients in their rehabilitation pathway, monitoring performance improvements, and physicians in the clinical evaluation of their patients.

The variability in the results concerning the exercises performed by the patients is linked to the different pathologies and to the different motor disabilities. For example, some patients do well in coordination exercises, but they do not have good finger control, reporting difficulties in the second exercise. Others, instead, can control their hands but cannot perform repeated wrist rotations in the third exercise.

The results of the first exercise (E1) express a significant statistical difference between study and control subjects in touching the capacitive sensor following the different frequencies. At both 60 bpm and 120 bpm, the study subjects perform fewer touch events in more time w.r.t. the control. This difference can be associated to the hand dysfunction caused by pathologies.

The second exercise (E2) shows study subjects took longer to draw the letters w.r.t. control subjects. Moreover, different execution times of the exercise are statistically significant in drawing letters *I* and *G*.

In the last exercise (E3), study subjects performed less prono-supination with an execution time longer than control subjects. Study subjects show difficulties in performing the entire exercise as a result of their motor disability.

The here proposed device has been tested in a rehabilitation clinical center and exercises have been performed also under the supervision of clinical experts to validate the instruments measurement performances. However, additional points need to be improved such as: (i) automatic control of back and front part of the hand in the exercises (see exercises E3); (ii) gravity control and measures acquisition modulation by means of gravity values. (iii) automatic uses in a domestic environment thus that patients could be guided during exercises and values acquisition. The reported issues could be considered as limitations for the current implemented version. However, the positive results in terms of measure reliability represent a positive validation that allow to consider the above reported issues as possible tuning phases for the improvements of the proposed device and release of an new version able to include improvements. Nevertheless, the complexity of the gesture underlies the analysis of the dexterity itself. In this scenario, this study has some limitations. first, this study had a small sample size without power statistics, although it is a proof-of-concept study; secondly, there is an underlying complexity of the gesture which is to be evaluated, but which guarantees the possibility of considering the manual dexterity itself. Thirdly, possible modifications or adjustments of the gesture related to different degrees of severity of the included neurological disorders could not be excluded. Fourth, the study needs further follow-up and possible considerations and postulations of a home-based approach.

## 5. Conclusions

The implemented device is able to support physicians and patients in rehabilitation by evaluating hand mobility affected by diseases. The programmable device has been tested by using three different types of exercises with study subjects and controls. Results show the reliability of the device in terms of data acquisition and its usability in physioterapy process for the characterization of subjects’ health status in upper limb pathologies. The execution of the proposed exercises by study and control subjects produces different results, confirming our hypothesis. In general, study subjects show lower performances in exercises execution (i.e., take more time or produce less touch events) w.r.t. control subjects. These results are statistically significant and could be associated to different hand motor abilities between the two groups. Finally, the device presents a cloud-based architecture allowing it to be used for the remote control of patients in home-based rehabilitation.

## Figures and Tables

**Table 1:** Values measured by the FSR402 sensor during the calibration measurements in the range of 0–20 kg for three different samples of known weight of 500 (*S*1), 1000 (*S*2) e 2000 (*S*3) grams, respectively. The means values are also reported in the last line.

Number of Measure	Detected Value for S1	Detected Value for S2	Detected Value for S3
1	20	34	50
2	21	35	52
3	25	36	54
4	26	41	68
5	27	42	70
6	28	43	71
7	29	44	72
8	30	45	74
Mean	25.75	40	63.87

**Table 2:** Clinical characteristics of the diseases contained in the considered dataset.

Disease	Description
Loeys-Dietz Syndrome	Rare genetic connective tissue disease
Multiple sclerosis	Chronic demyelinating neurodegenerative disease affecting any type of nerve
Omarthrosis	Degenerative disease affecting the shoulder joint, causing the wear of the articular cartilage
Parkinson	Neurodegenerative disese with mobility impairment
Rhizoarthrosis	Arthrosis localized at the level of the trapezio-metacarpal joint
Cerebellar Ataxia	Cerebellum damage causing impairment in motor skills
Limb-girdle muscular dystrophy	Diseases characterized by weakness and wasting of the muscles in the arms and legs
Stroke	Cerebrovascular event associated with different types of movement disorders
Hirayama Syndrome	Cervical myelopathy presenting spinal muscular atrophy of the distal upper limbs

**Table 3:** E1 exercise type, performed by the whole set of subjects analyzed, for both metronome at 60 and 120 bpm, measured in terms of the absolute value of the difference between the mean counts of T events and the expected counts (*Normalized count* column) and of the absolute value of the difference between the mean time for a single T event and the expected time (*Normalized time* column). Results are reported in terms of mean and standard deviation values both for study and control subjects. Furthermore, the statistical significance of the difference between the same measures is reported in the bottom row.

Experiments	Control	Study	*p*-Value
Normalized count at 60 bpm	0.72 ± 0.93	1.58 ± 1.22	<0.01
Normalized time at 60 bpm	30.16 ± 25.82	47.96 ± 29.15	<0.01
Normalized count at 120 bpm	0.67 ± 1.00	6.50 ± 5.84	<0.01
Normalized time at 120 bpm	9.89 ± 10.00	21.57 ± 12.81	<0.01

**Table 4:** E2 exercise type, performed by drawing letters I, V, G and M on the sensor surface. Results are reported as mean values (with standard deviation) of time taken by both study and control subjects to draw each letter. The values are expressed in milliseconds (ms). Moreover, *p*-values from T-test are also reported in the bottom row to express the statistical significance of the difference between the same measurements for both study and control groups.

Experiments	Control	Study	*p*-Value
Letter I	1127.18 ± 1286.60	2109.98 ± 977.15	<0.01
Letter V	2056.48 ± 1398.00	2954.70 ± 1915.17	<0.01
Letter G	2426.98 ± 1284.80	4130.80 ± 1763.47	<0.01
Letter M	2522.55 ± 1388.21	5245.03 ± 2421.81	<0.01

**Table 5:** E3 exercise type, performed by a series of prono-supination events. Results are expressed as average count and average time in milliseconds (ms) of P-S events both for study and control subjects. T-tes results are reported in terms of *p*-value.

Experiments	Control	Study	*p*-Value
Avg count of P-S events	37.60 ± 8.81	20.73 ± 5.56	<0.01
Avg Time for a P-S event [ms]	575.08 ± 131.59	1203.00 ± 433.73	<0.01

## Data Availability

Data is property of a public structure and can be furnished on requests.
